# Point-of-Care Diagnostic Technologies for Antimicrobial Resistance: Principles, Platforms, Clinical Impact, and Future Directions

**DOI:** 10.3390/diagnostics16081239

**Published:** 2026-04-21

**Authors:** Nahed N. Mahrous, Mohannad M. Fallatah, Rawan A. Fitaihi, Hala Aldahshan, Areej A. Alhhazmi, Samiyah Al-Khaldi, Hussam Fallatah, Abdulmajeed A. Althobaiti, Abdulaziz Saleh Alkhoshaiban, Jawaher Alguraini, Esraa A. Aldkheil, Yahya F. Jamous

**Affiliations:** 1 Department of Biological Sciences, College of Science, University of Hafr Al-Batin, Hafr Al-Batin 39524, Saudi Arabia; nnmahrous@uhb.edu.sa; 2 Advanced Diagnostics and Therapeutics Institute, Health Sector, King Abdulaziz City for Science and Technology (KACST), Riyadh 12354, Saudi Arabia; 3 Department of Pharmaceutics, College of Pharmacy, King Saud University, Riyadh 11451, Saudi Arabia; rfitaihi@ksu.edu.sa; 4 Department of Clinical Laboratory Sciences, College of Applied Medical Sciences, King Saud University, Riyadh 11451, Saudi Arabia; haldahshan@ksu.edu.sa; 5 Department of Clinical Laboratory Sciences, College of Applied Medical Sciences, Taibah University, Medina 42353, Saudi Arabia; ahazmib@taibahu.edu.sa; 6 Applied Genomics Technologies Institute, Health Sector, King Abdulaziz City for Science and Technology (KACST), Riyadh 11442, Saudi Arabia; salkhaldi@kacst.gov.sa; 7 Institute of Waste Management and Recycling Technologies, King Abdulaziz City for Science and Technology (KACST), Riyadh 12354, Saudi Arabia; hfallatah@kacst.gov.sa; 8 Pharmacy Department, College of Pharmacy, Nursing and Medical Sciences, Riyadh Elm University, Riyadh 11681, Saudi Arabia; abdulmajeed.althobaiti@riyadh.edu.sa; 9 Unit of Scientific Research, Applied College, Qassim University, Qassim 52571, Saudi Arabia; a.alkhoshaiban@qu.edu.sa; 10 Health Holding Company, Riyadh 13215, Saudi Arabia; jawaher.alguraini@health.sa; 11 Bioengineering Institute, Health Sector, King Abdulaziz City for Science and Technology (KACST), Riyadh 11442, Saudi Arabia; ealdkheil@kacst.gov.sa; 12 Wellness and Preventive Medicine Institute, Health Sector, King Abdulaziz City for Science and Technology (KACST), Riyadh 11442, Saudi Arabia

**Keywords:** point-of-care diagnostics, antimicrobial resistance, rapid antimicrobial susceptibility testing, biosensors, microfluidics, antimicrobial stewardship

## Abstract

Antimicrobial resistance (AMR) is an ever-growing threat to global healthcare. It is largely driven by delayed or inadequate pathogen identification and antimicrobial susceptibility testing in routine clinical workflows. By the time the clinician receives results to guide treatment from traditional culture-based diagnostics, several days may have elapsed, leading to the use and potential over-prescription of broad-spectrum antibiotics and the development of resistant pathogens. A rapid and clinically actionable diagnostic approach at the clinical point of care (POC) may help address this gap. This review examines current and emerging POC diagnostic technologies for AMR and outlines the fundamental principles and mechanistic classifications of POC diagnostic technologies. These include phenotypic, genotypic, immunological, and biosensor-based approaches. A critical overview of key technological platforms, including rapid phenotypic antimicrobial susceptibility testing (AST), microfluidics and isothermal nucleic acid amplification (e.g., LAMP and RPA), CRISPR-based diagnostics, nanomaterial-enhanced biosensors, and mobile-integrated systems is provided. The impact of POC diagnostics on antimicrobial stewardship, time to appropriate therapy, and patient outcomes in primary care settings, hospitals, intensive care units, and resource-limited settings is presented and discussed. In addition to clinical implementation challenges, this review considers the issues of analytical performance, workflow, regulatory pathways, cost, and implementation readiness. In addition, it outlines key trends regarding digital integration, surveillance, workforce training, and policy frameworks. Overall, the review outlines the role of POC diagnostics in enhancing antimicrobial response surveillance and the global fight against AMR. Among emerging platforms, rapid phenotypic AST, microfluidic and isothermal-based assays, CRISPR-based diagnostics, and integrated biosensor systems show the greatest potential for near-term clinical impact; however, widespread implementation remains constrained by challenges related to clinical validation, cost, workflow integration, and alignment with antimicrobial stewardship frameworks.

## 1. Introduction

Antimicrobial resistance (AMR) constitutes a significant global public health threat in the 21st century, undermining decades of progress in infectious disease prevention and treatment. Resistant bacterial infections contribute to increased morbidity, prolonged hospitalization, higher healthcare costs, and elevated mortality rates worldwide. Recent global burden analyses estimate that bacterial AMR was directly responsible for over 1 million deaths in 2019, with even greater numbers resulting from indirect effects such as treatment failure and complications. The slow pace of new antibiotic development further intensifies the problem, causing existing antimicrobial agents to become progressively less effective against common pathogens [1,2,3,4]. Conventional microbiological diagnostics primarily depend on culture-based methods followed by antimicrobial susceptibility testing (AST), a process that typically requires 24 to 72 h or longer to yield actionable results. During this period, clinicians frequently initiate empirical therapy, often using broad-spectrum antibiotics to reduce the risk of clinical deterioration. Although empirical treatment can be lifesaving, it is also linked to inappropriate antibiotic use, unnecessary exposure to broad-spectrum agents, and increased selective pressure on resistant strains. Therefore, the lack of rapid, reliable diagnostic information at the point of clinical decision-making directly contributes to the emergence and spread of AMR [5,6,7,8]. This is further supported by recent evidence demonstrating that rapid diagnostics play a critical role in enabling antimicrobial stewardship and optimizing antibiotic use [9]. Importantly, the challenge extends beyond diagnostic delay to the lack of timely, actionable AMR information at the point of clinical decision-making, which limits the ability to optimize therapy and effectively support antimicrobial stewardship. Addressing AMR requires a multifaceted approach that extends beyond diagnostics alone, including the development of effective vaccines, optimization of antibiotic therapies, and implementation of antimicrobial stewardship strategies. Vaccination, in particular, plays a critical role in reducing infection incidence and antibiotic consumption, thereby limiting the selective pressure that drives resistance emergence [10].

Point-of-care (POC) diagnostics address the diagnostic gap by enabling rapid testing at or near the site of patient care. In contrast to centralized laboratory assays, POC diagnostics are designed to deliver clinically relevant results within a timeframe that supports immediate therapeutic decisions. The World Health Organization (WHO) has emphasized the importance of these diagnostics through guidelines specifying desirable characteristics for decentralized testing, particularly in resource-limited settings (WHO ASSURED criteria). These criteria emphasize that POC tests should be Affordable, Sensitive, Specific, User-friendly, Rapid and robust, Equipment-free, and Deliverable to end users, particularly in resource-limited settings.

In the context of AMR, POC diagnostics can reduce time-to-result, guide targeted antimicrobial therapy, decrease unnecessary antibiotic prescriptions, and support antimicrobial stewardship initiatives [11,12,13,14]. Beyond individual patient management, POC diagnostics exert broad impacts on healthcare systems and public health. Rapid identification of pathogens and resistance determinants can enhance infection prevention and control, facilitate early outbreak detection, and improve surveillance of resistance trends at local, national, and global levels. In primary care and emergency settings, POC tests provide objective evidence to inform clinical decision-making and strengthen clinician–patient communication [15]. Furthermore, advances in digital connectivity increasingly enable POC devices to integrate with electronic health records and surveillance networks, thereby extending their impact beyond the immediate point of testing [16].

This review provides a comprehensive analysis of current and emerging POC diagnostic technologies for antimicrobial resistance. It examines the fundamental principles underlying POC AMR diagnostics, surveys phenotypic, genotypic, immunological, and biosensor-based platforms, and discusses their clinical applications, limitations, and future directions. Unlike previous reviews that often focus primarily on specific technological approaches or analytical performance, this work adopts an integrated perspective that links technological innovation with clinical implementation, translational readiness, and real-world impact on antimicrobial stewardship and healthcare systems. By combining technological, clinical, and policy dimensions, this review aims to provide a comprehensive framework for evaluating and guiding the deployment of POC AMR diagnostics across diverse healthcare settings [17,18,19,20,21].

## 2. Fundamentals of Point-of-Care Diagnostics for Antimicrobial Resistance

**Figure 1:**
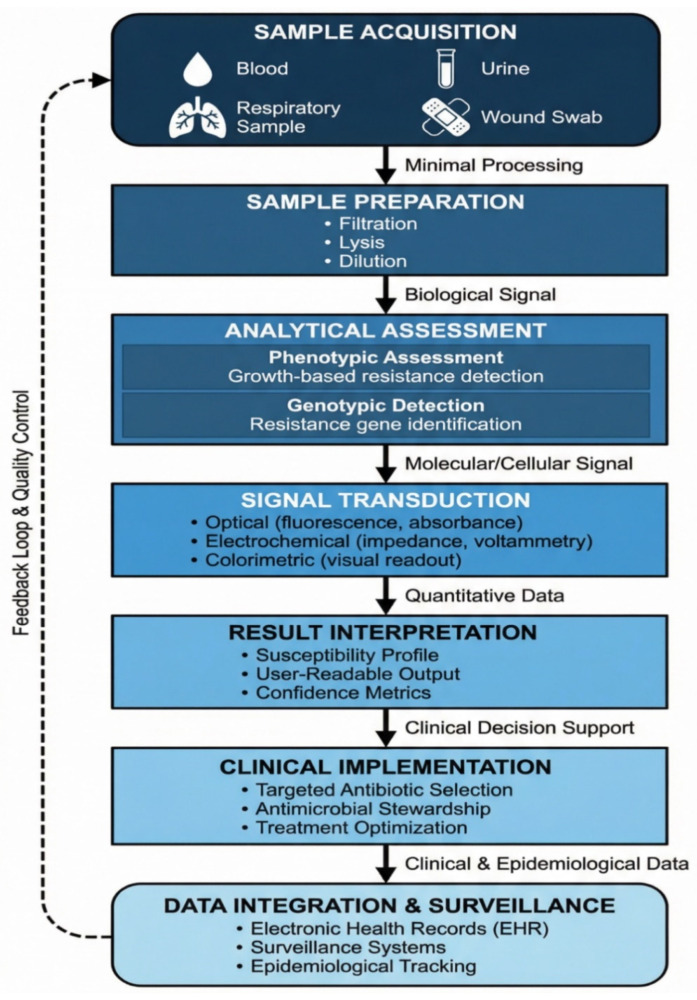
Conceptual workflow of point-of-care diagnostics for antimicrobial resistance (AMR). The workflow depicts sequential stages from sample acquisition (e.g., blood, urine, respiratory samples, and wound swabs) and minimal sample preparation (filtration, lysis, and dilution) to analytical assessment using phenotypic (growth-based resistance detection) and genotypic (resistance gene identification) approaches. Molecular or cellular signals are converted through signal transduction modalities, including optical, electrochemical, and colorimetric readouts, generating quantitative data for result interpretation. The interpreted outputs support clinical decision-making, enabling targeted antibiotic selection, antimicrobial stewardship, and treatment optimization. Integrated feedback loops link clinical implementation with data integration and surveillance systems, including electronic health records and epidemiological tracking.

POC diagnostics are characterized by their capacity to deliver actionable diagnostic information at or near the site of patient care, without dependence on centralized laboratory infrastructure. For AMR, POC diagnostics must address both pathogen identification and detection of antimicrobial susceptibility or resistance determinants within a clinically relevant timeframe. This dual requirement distinguishes AMR diagnostics from many other infectious disease tests and introduces unique technical and operational challenges for assay design. POC diagnostics prioritize speed, simplicity, and usability. Tests are typically designed for minimal sample preparation, limited hands-on time, and straightforward result interpretation by non-specialist users. For AMR applications, these features must be balanced with the need for analytical accuracy, as false-negative or false-positive resistance results can have significant clinical consequences. Consequently, POC AMR diagnostics must achieve high sensitivity and specificity while maintaining robustness across diverse environmental and operational conditions [11,22]. From a clinical perspective, these performance requirements are critical because inaccurate or delayed resistance information can directly influence antimicrobial selection, patient outcomes, and stewardship decisions. The general workflow and decision-making structure of POC diagnostics for AMR are summarized in Figure 1, which presents a conceptual workflow and decision-making framework for POC diagnostics in antimicrobial resistance, illustrating the progression from sample acquisition and analytical assessment to clinical decision-making, antimicrobial stewardship, and surveillance integration. Based on these functional requirements, current POC AMR diagnostics can be mechanistically classified into two major categories.

Mechanistically, POC AMR diagnostics can be broadly categorized into phenotypic and genotypic approaches. Phenotypic diagnostics assess the functional response of bacteria to antimicrobial agents, typically by measuring growth inhibition, metabolic activity, or viability in the presence of antibiotics. These methods directly evaluate antimicrobial susceptibility and are generally independent of the specific resistance mechanisms involved. However, phenotypic assays often require viable organisms and may be constrained by bacterial growth rates, which can limit their speed even in miniaturized or accelerated formats. In contrast, genotypic diagnostics detect specific genetic determinants associated with resistance, such as resistance genes, mutations, or mobile genetic elements. These methods can provide rapid results and are applicable to non-viable organisms [23,24]. Nevertheless, they depend on existing knowledge of resistance mechanisms and may not identify novel or phenotypically expressed resistance. Importantly, the presence of a resistance gene does not always correspond to clinical resistance, underscoring the need for careful interpretation and, in some cases, supplementary phenotypic confirmation [25]. The key conceptual distinctions between phenotypic and genotypic POC AMR diagnostics are summarized in Table 1. This comparison highlights the complementary strengths and inherent limitations of phenotypic and genotypic approaches in POC settings, particularly with respect to clinical interpretability, turnaround time, and resistance mechanism coverage.

The operational context is a critical consideration in the design and implementation of POC AMR diagnostics. In high-income healthcare settings, integration with electronic health records, antimicrobial stewardship programs, and laboratory information systems is increasingly standard. In low- and middle-income countries (LMICs), where laboratory infrastructure may be limited, POC diagnostics must prioritize affordability, robustness, and minimal reliance on external equipment or cold-chain logistics. These contextual factors influence not only technology selection, but also regulatory approval, reimbursement, and long-term sustainability. To guide the development and evaluation of POC diagnostics, the WHO established the ASSURED criteria: Affordable, Sensitive, Specific, User-friendly, Rapid and Robust, Equipment-free, and Deliverable to end users [26]. More recent frameworks have identified additional requirements, such as real-time connectivity and ease of specimen collection. Although no single diagnostic platform fulfills all these criteria, they provide a valuable benchmark for assessing the suitability of POC AMR diagnostics across diverse clinical and geographical contexts. The primary challenge in POC diagnostics for AMR is to achieve an optimal balance among analytical performance, operational simplicity, and clinical relevance. Technologies that achieve this balance have the potential to transform antimicrobial prescribing practices, enhance surveillance systems, and play a central role in global efforts to contain AMR [27].

## 3. Current Point-of-Care Technologies for Antimicrobial Resistance Detection

Building on the foundational principles of POC diagnostics for antimicrobial resistance, current POC technologies translate these concepts into practical diagnostic platforms that aim to provide rapid, clinically actionable information while minimizing dependence on centralized laboratory infrastructure. These technologies encompass a wide spectrum of analytical strategies that vary in complexity, turnaround time, and operational requirements, but share a common goal of enabling timely antimicrobial decision-making at or near the site of patient care. Importantly, the design of POC AMR technologies reflects a balance between analytical performance and real-world feasibility, particularly in settings where access to conventional microbiology laboratories is limited or delayed [24,28].

Recent advances in miniaturization, microfabrication, and biosensing have accelerated the development of POC platforms capable of integrating sample processing, analysis, and result interpretation into compact systems. These innovations have expanded the scope of POC diagnostics beyond simple pathogen detection to include functional and molecular assessments of antimicrobial susceptibility. As a result, POC technologies are increasingly positioned not only as rapid screening tools, but also as integral components of antimicrobial stewardship strategies and surveillance frameworks across diverse healthcare environments [25,29].

### 3.1. Phenotypic Point-of-Care Approaches

Phenotypic POC approaches are centered on the direct measurement of bacterial behavior in the presence of antimicrobial agents, thereby providing functional susceptibility information that closely reflects the in vivo response to therapy. By assessing growth inhibition, metabolic activity, or cell viability, these approaches capture the combined effects of all expressed resistance mechanisms, regardless of their genetic basis. This characteristic distinguishes phenotypic POC assays from genotypic methods and underpins their continued clinical relevance, particularly in scenarios where resistance mechanisms are complex, inducible, or incompletely characterized [23,25].

Despite their conceptual alignment with clinical decision-making, conventional phenotypic AST methods are constrained by prolonged incubation times and reliance on laboratory infrastructure. Phenotypic POC strategies seek to overcome these limitations by accelerating bacterial response detection through assay miniaturization, optimized growth conditions, and alternative readouts that enable earlier discrimination between susceptible and resistant phenotypes. These innovations aim to retain the interpretability of traditional AST while substantially reducing time-to-result, thereby enhancing their utility in acute care and decentralized testing settings [25,30].

#### 3.1.1. Rapid Antimicrobial Susceptibility Testing

Rapid phenotypic AST constitutes a cornerstone of POC strategies for AMR, as it focuses on compressing conventional growth-based AST workflows into clinically actionable timeframes without compromising interpretability. Rather than relying solely on visible colony formation, rapid AST platforms employ early indicators of bacterial response, including changes in cell morphology, metabolic flux, nucleic acid synthesis, or membrane integrity, to infer susceptibility outcomes at earlier time points. These approaches enable susceptibility assessment within hours, or in some cases minutes, compared with the multi-day timelines associated with standard laboratory methods [23,24,25].

The clinical value of rapid AST lies in its potential to inform antimicrobial optimization at a stage when therapeutic decisions remain modifiable. Earlier availability of susceptibility data can facilitate timely escalation or de-escalation of therapy, reduce unnecessary exposure to broad-spectrum agents, and support antimicrobial stewardship interventions, particularly in high-risk settings such as bloodstream infections and intensive care units. However, the development of rapid AST platforms also introduces technical challenges, including maintaining analytical accuracy, ensuring reproducibility across variable bacterial loads, and integrating results into existing clinical workflows. Addressing these challenges remains critical for the broader adoption of rapid phenotypic AST within POC environments [27,28,29].

#### 3.1.2. Microfluidics-Based Growth Inhibition Assays

Microfluidic technology involves the manipulation of fluids within networks of microscale channels, typically handling volumes in the microliter to nanoliter range. At these scales, the fluid flow is predominantly laminar, and diffusion governs mixing, allowing highly controlled reaction environments. Devices are commonly fabricated from materials such as polydimethylsiloxane (PDMS), glass, or thermoplastics, which are selected for their optical transparency, biocompatibility, and ease of integration with detection modules [31].

By integrating sample preparation, amplification, and detection on a single chip, microfluidic systems can substantially shorten diagnostic workflows and minimize reagent consumption. Ref. [30] demonstrated a microfluidic PCR platform capable of detecting bacterial clusters and resistance genes within approximately 70 min, thus emphasizing the potential of on-chip amplification for rapid AMR diagnostics [31]. Other studies have shown that microfluidic devices can be used to screen antibiotic combinations at the single-cell level, characterize heterogeneous resistance phenotypes in real-time, and perform multiplexed assays in parallel reaction chambers [32].

Microfluidics also enables seamless coupling with other analytical modalities. For example, microfluidic mass spectrometry systems have been used for rapid AST, while microfluidic chips have been combined with optical or electrochemical biosensors to enhance sensitivity and allow high-throughput screening [30,31]. In low-resource settings, microfluidic platforms can be designed to operate with minimal external infrastructure, providing decentralized tools for pathogen and AMR surveillance.

Despite their promise, microfluidic platforms face several technical and practical barriers that limit their widespread adoption in routine bacterial and AMR diagnostics. One important challenge is related to flow dynamics. Systems that rely on narrow or submicron channels for bacterial capture are constrained by low volumetric flow rates. This limits the sample throughput and may be inadequate for processing clinically relevant volumes, particularly for blood or environmental samples with low bacterial loads [27,32]. Maintaining stable channel geometries often requires precise backpressure control, as deviations can deform channels or dislodge captured bacteria, leading to a loss of sensitivity and reproducibility.

The material properties further constrain device performance. Hydrogels such as alginate are attractive for cell encapsulation but often lack the mechanical robustness and long-term stability required for complex flow regimes. Additionally, their limited cell-adhesion properties can impair bacterial capture. Regenerated fibers, such as silk, may not fully reproduce the mechanical and biochemical characteristics of their natural counterparts, and slow fiber spinning speeds hinder scalable manufacturing [33]. While complex three-dimensional architectures enhance multiplexing capacity, they also increase fabrication costs and complicate mass production.

Surface-based immobilization strategies have some other limitations. For examples, although coatings such as chitosan can improve bacterial adhesion, motile strains with active flagella may poorly adhere to the surface, necessitating fixation steps that alter cell physiology and narrow the range of detectable species [27]. Workflows based on multiplexed fluorescence in situ hybridization (FISH), such as 16S rRNA-FISH, typically require sequential reagent infusions, extend assay times, and make high-throughput or urgent analyses challenging [32,34]. Gram-positive bacteria, which are less susceptible to permeabilization by agents such as lysozyme, can be particularly difficult to profile, thereby reducing the overall classification accuracy across mixed communities.

Portability and costs are important considerations. Many microfluidic platforms depend on external imaging systems, such as high-resolution fluorescence microscopes or confocal scanners, which substantially increase capital costs and limit their deployment in low-resource settings. When bacterial concentrations are low, samples are polymicrobial, pre-enrichment, or culture may still be required, partially negating the time advantages of on-chip analysis [32].

Furthermore, DNA or protein-based microfluidic assays cannot easily distinguish viable and non-viable cells, restricting their use when live bacterial detection is required, such as in AST. Emerging approaches, including microfluidic paper-based analytical devices (µPADs) and micro-total analysis systems (µTAS), are attractive because of their low cost and ease of use; however, they often depend on cold-chain storage of reagents and stable power supplies, which can be problematic in remote settings [35]. Many prototypes remain at the proof-of-concept stage, and further optimization and clinical validation are needed before widespread implementation. Despite these challenges, ongoing advances in microfabrication, integrated sensing, and data analysis continue to strengthen the potential of microfluidic platforms as scalable solutions for rapid phenotypic AMR testing. In future developments, achieving an optimal balance among assay duration, sensitivity, multiplexing capacity, and operational complexity will be essential to ensure that microfluidic platforms are appropriately aligned with specific applications in clinical diagnostics, environmental surveillance, and food safety.

### 3.2. Genotypic Point-of-Care Approaches

Genotypic POC approaches aim to rapidly identify pathogens and resistance determinants at the molecular level, providing early diagnostic information that can guide antimicrobial selection before phenotypic susceptibility results become available.

#### 3.2.1. Isothermal Amplification and PCR Technologies

**Figure 2:**
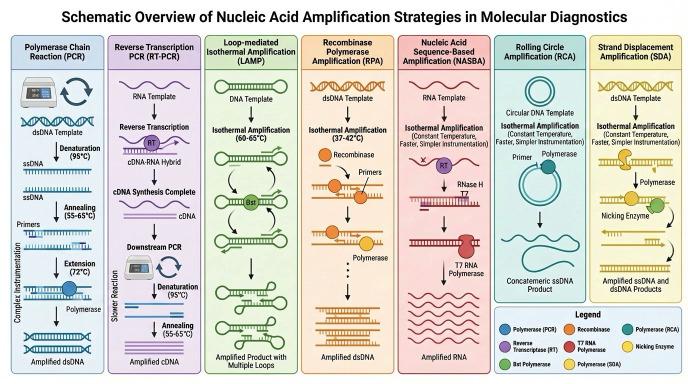
Schematic overview of nucleic acid amplification strategies used in molecular diagnostics. The figure compares conventional polymerase chain reaction (PCR), which relies on thermocycling steps of denaturation, annealing, and extension, with representative IAT, including LAMP, RPA, and SDA. While PCR requires precise temperature cycling, IATs enable continuous amplification at a constant temperature through distinct enzymatic mechanisms, allowing faster reaction times and simpler instrumentation. These characteristics make isothermal methods particularly suitable for point-of-care diagnostic applications.

Rapid and accurate detection of bacterial pathogens and AMR determinants are essential to improve patient outcomes and slow the spread of resistance. Nucleic acid amplification methods have become a cornerstone of modern diagnostics as they allow the direct detection of pathogen-specific genetic material and resistance genes with high analytical sensitivity. Conventional polymerase chain reaction (PCR) remains the reference method, but its reliance on repeated temperature cycling, relatively long turnaround times, and the need for sophisticated equipment and trained personnel limit its deployment at the POC (Figure 2). These limitations have driven the development of isothermal amplification methods specifically optimized for decentralized and near-patient testing environments.

Isothermal amplification technology (IAT) encompasses different methods that amplify nucleic acids at a constant temperature (Figure 2), thereby eliminating the requirement for thermocycling. These assays typically rely on three core components: a nucleic acid template (DNA or RNA), sequence-specific primers and enzymes that combine polymerase activity with strand displacement or recombinase functions [36,37]. Operating at a single temperature simplifies instrumentation, reduces energy consumption, and allows the use of portable heaters. Consequently, IAT platforms are particularly attractive for decentralized testing in primary care, community clinics, and low-resource settings [36,37].

Compared to PCR, IAT offers several advantages. Many formats are tolerant to crude samples, thereby reducing the need for extensive nucleic acid extraction. Reaction times are often markedly shorter, with visible or instrument-read outputs generated within 10–60 min. In addition, several IAT methods can accept double-stranded DNA (dsDNA), single-stranded DNA (ssDNA), or RNA as inputs, broadening their diagnostic utility in both DNA and RNA pathogen detection [38,39]. These features make IAT highly compatible with POC devices and with complementary technologies, such as microfluidics and biosensors.

Despite these benefits, some important limitations remain. Primer design rules are more complex than those for PCR, and there is limited consensus and standardization across platforms. Industrial-scale production of optimized reagents and ready-to-use kits is still evolving, and some methods are highly susceptible to carry-over contamination due to the large amount of amplicons generated [36]. Moreover, achieving robust multiplexing while preserving sensitivity and specificity remains technically challenging. These general advantages and constraints have shaped the development and clinical adoption of specific isothermal amplification platforms, which differ in complexity, robustness, and suitability for POC deployment.

##### Loop-Mediated Isothermal Amplification

Loop-mediated isothermal amplification (LAMP) is one of the most widely adopted and clinically explored isothermal nucleic acid amplification platforms for POC diagnostics. First described by Notomi et al. in 2000, LAMP is a well-established technique that enables rapid and highly specific DNA amplification under isothermal conditions [40].

LAMP employs a set of four to six primers that recognize six to eight distinct regions in the target sequence. The canonical primer set comprises two outer primers (F3 and B3), two inner primers (FIP and BIP) that drive exponential amplification and optional loop primers that bind loop regions of the initial amplicons to accelerate the reaction [41]. Strand-displacing DNA polymerase, typically Bst polymerase, extends primers at a constant temperature (usually 60–65 °C), generating characteristic cauliflower-like DNA structures with multiple loops.

This primer architecture underpins the high analytical specificity of the LAMP assay and enables efficient amplification. Up to 10^9^ copies of the target can be generated within approximately one hour, significantly exceeding the amplification achieved by standard PCR within a similar timeframe [42]. LAMP reactions can be read by turbidity, fluorescence, and color change using pH-sensitive or metal-indicator dyes or lateral-flow strips, allowing both instrument-based and visually interpretable formats [40,42,43].

LAMP assays are relatively simple to set up, often using pre-formulated “master mixes”, which require only the addition of primers and samples. Minimal hands-on time and straightforward detection have led to broad adoption in infectious disease diagnostics, including bacterial, viral, and parasitic agents, as well as in applications such as single nucleotide polymorphism (SNP) genotyping, DNA methylation analysis, and other genetic variation analyses [42,43,44]. The robustness of LAMP to common inhibitors and its compatibility with crude clinical specimens make it particularly attractive for POC AMR testing where rapid triage and treatment decisions are required.

##### Recombinase Polymerase Amplification

Recombinase polymerase amplification (RPA) is a low-temperature isothermal amplification method developed in 2006 that harnesses recombination and strand-displacement processes to drive target amplification [43,44]. RPA reactions typically operate between 37 °C and 42 °C and use three principal proteins: a recombinase that forms complexes with primers and facilitates homology-directed invasion of double-stranded templates, single-stranded DNA-binding proteins that stabilize the displaced strand, and a strand-displacing polymerase that extends the primers without the need for thermal denaturation [45].

A major advantage of the RPA is its speed. Detectable amplicons can be produced in as little time as 10–20 min, and amplification can often proceed at ambient or body temperature, making RPA highly suitable for portable and field-deployable diagnostics [46,47]. Primer design is generally less complex than that for LAMP, and reactions can be configured for real-time fluorescence, endpoint fluorescence, or lateral-flow read-outs. RPA has been applied for the detection of a broad range of microorganisms, including bacteria, fungi, viruses, and parasites, as well as genetically modified organisms and resistance genes [46,47].

By addressing several limitations of culture-based diagnostics, RPA enables rapid and sensitive detection without the need for prolonged incubation times required for microbial growth. Its short turnaround time positions RPA as a strong candidate for integration into POC platforms targeting AMR in both high-income and low-income settings.

##### Strand Displacement Amplification

Strand displacement amplification (SDA) is an isothermal technique that couples primer-initiated DNA synthesis with the action of restriction endonucleases and strand-displacing polymerases [41]. The reaction is initiated by primer annealing to the target sequence under isothermal conditions. Two primers (S1 and S2) contain target-binding regions and recognition sites for a restriction enzyme (for example, HincII), whereas a second pair of primers (B1 and B2) provides additional binding sites to drive exponential amplification [41].

Following incorporation into the nascent DNA strands, the restriction enzyme introduces nicks at its recognition sites under non-denaturing conditions. A strand-displacing polymerase then extends from the 3′-OH at the nick, displacing the downstream DNA and generating new templates for subsequent rounds of nicking and extension. The result is an isothermal amplification process that can be coupled with diverse downstream detection methods, including fluorescence, chemiluminescence, and electrochemical readouts [48,49,50,51].

Recent innovations have further expanded the potential of SDA-based assays. Wang et al. (2020) developed an electrochemical biosensor integrating NsbI-mediated SDA with a four-way DNA junction for ultrasensitive detection of the PIK3CA H1047R mutation, achieving a detection limit of 0.001% [52]. SDA has also been used for miRNA detection and other low-abundance nucleic acid targets, highlighting its relevance in precision oncology and clinical genetics [38,50]. Together, these isothermal amplification strategies illustrate the diversity of molecular approaches available for POC AMR diagnostics, while also highlighting trade-offs between speed, complexity, and clinical implementation. These properties also support the potential adaptation of SDA-based platforms for AMR detection.

##### Nucleic Acid Sequence-Based Amplification

Nucleic acid sequence-based amplification (NASBA) is an isothermal amplification technique specifically designed for the detection of RNA targets and operates at a constant temperature, typically around 41 °C. Originally developed for the amplification of viral and bacterial RNA, NASBA enables highly sensitive detection of transcriptionally active targets without the need for thermal cycling. The method relies on the coordinated activity of three enzymes: reverse transcriptase, RNase H, and T7 RNA polymerase, which together drive continuous RNA amplification under isothermal conditions [53,54].

In a typical NASBA reaction, reverse transcriptase synthesizes complementary DNA (cDNA) from the target RNA using a primer containing a T7 promoter sequence. RNase H subsequently degrades the RNA strand of the RNA–DNA hybrid, allowing synthesis of a second DNA strand. The resulting double-stranded DNA template serves as a substrate for T7 RNA polymerase, which produces multiple RNA transcripts that re-enter the amplification cycle, enabling rapid and exponential signal generation [54,55].

A key advantage of NASBA is its high specificity for RNA, making it particularly suitable for detecting viable microorganisms and actively expressed resistance genes. Detection of NASBA amplicons can be achieved using real-time fluorescence, molecular beacons, electrochemical sensors, or lateral-flow formats, supporting both laboratory-based and POC implementations [55,56]. NASBA has been widely applied in viral diagnostics, including HIV, influenza, and SARS-related viruses, as well as in bacterial pathogen detection and AMR surveillance [54].

The isothermal nature of NASBA, combined with its ability to selectively amplify RNA, positions this technique as a valuable platform for POC diagnostics targeting antimicrobial resistance. By enabling rapid identification of active resistance determinants without extensive sample processing, NASBA offers a complementary approach to DNA-based isothermal amplification methods in decentralized and resource-limited settings.

A comparative evaluation of major isothermal amplification technologies highlights important differences in their suitability for POC AMR diagnostics. Loop-mediated isothermal amplification (LAMP) is characterized by high analytical specificity due to its multi-primer design and has achieved a high level of clinical validation among isothermal methods [40,42,43,44]. Recombinase polymerase amplification (RPA) offers the fastest amplification kinetics and operates at near-physiological temperatures, making it particularly attractive for portable and field-deployable diagnostic platforms [43,44,45,46,47]. Strand displacement amplification (SDA) provides high sensitivity through enzyme-mediated strand displacement but is relatively more complex and less widely adopted in clinical settings [48,49,50,51,52]. In contrast, nucleic acid sequence-based amplification (NASBA) is uniquely suited for RNA detection and enables identification of transcriptionally active resistance genes, offering potential advantages in distinguishing viable pathogens [53,54,55,56]. Despite these differences, all platforms share common challenges, including susceptibility to contamination, limited multiplexing capacity, and the need for standardized assay design and robust clinical validation [48,49,50,51,52,57]. These trade-offs should be carefully considered when selecting appropriate technologies for decentralized AMR diagnostics.

#### 3.2.2. CRISPR-Based Diagnostics

CRISPR-based diagnostics represent a transformative class of nucleic acid detection technologies that leverage the programmable sequence recognition capabilities of CRISPR-associated (Cas) enzymes. Originally developed as adaptive immune systems in bacteria and archaea, CRISPR–Cas systems have been repurposed for highly specific molecular diagnostics capable of detecting DNA or RNA targets with single-base resolution. Their ability to combine target recognition with signal amplification has positioned CRISPR-based platforms as powerful tools for POC diagnostics, particularly in infectious disease detection and AMR surveillance [57,58].

Most CRISPR diagnostic platforms rely on Cas enzymes with collateral (trans-cleavage) activity, such as Cas12 and Cas13. Upon binding to a specific target sequence via a guide RNA (gRNA), the activated Cas enzyme nonspecifically cleaves nearby reporter molecules, generating a detectable signal. Cas12 systems are typically used for DNA targets and produce single-stranded DNA cleavage, whereas Cas13 systems target RNA and cleave single-stranded RNA reporters. This collateral cleavage mechanism enables signal amplification independent of target quantity and can be coupled with fluorescence, colorimetric, electrochemical, or lateral-flow readouts [57,58].

CRISPR-based diagnostics are often integrated with upstream isothermal amplification methods such as LAMP, RPA, or PCR to enhance sensitivity and enable detection of low-abundance targets. Platforms such as SHERLOCK (Specific High Sensitivity Enzymatic Reporter Unlocking) and DETECTR (DNA Endonuclease-Targeted CRISPR Trans Reporter) have demonstrated attomolar-level sensitivity and high specificity for viral, bacterial, and genetic targets, including single nucleotide polymorphisms associated with AMR [57,58,59].

In the context of AMR diagnostics, CRISPR-based systems offer distinct advantages, including precise discrimination of resistance genes, point mutations, and allelic variants that confer antimicrobial resistance. Their compatibility with rapid, low-temperature workflows and minimal instrumentation makes them particularly attractive for decentralized testing in resource-limited and field settings. As ongoing developments continue to simplify assay design, reduce cost, and enable multiplexing, CRISPR-based diagnostics are expected to play an increasingly important role in next-generation POC platforms for AMR detection and surveillance.

#### 3.2.3. DNA Microarrays and Cartridge-Based Platforms

DNA microarrays and cartridge-based platforms represent established molecular diagnostic approaches that enable the parallel detection of multiple genetic targets within a single assay. DNA microarrays rely on the hybridization of labeled nucleic acid targets to immobilized oligonucleotide probes arranged in a high-density array format, allowing simultaneous interrogation of numerous genes, mutations, or resistance determinants. This multiplexing capability has made microarrays particularly valuable for comprehensive AMR profiling, where multiple resistance genes or allelic variants may coexist within a single pathogen [59].

In AMR diagnostics, DNA microarrays have been widely applied for the detection of resistance-associated genes, point mutations, and gene expression signatures across bacterial and viral pathogens. Their ability to provide broad resistance landscapes rather than single-target readouts offers a distinct advantage over conventional singleplex assays. However, traditional microarray workflows often require centralized laboratory infrastructure, multiple processing steps, and skilled personnel, which can limit their applicability in decentralized or POC settings [59].

To address these limitations, cartridge-based molecular platforms have emerged as integrated diagnostic systems that combine sample preparation, nucleic acid amplification, detection, and result interpretation within closed, disposable cartridges. These systems minimize hands-on time and reduce contamination risk while delivering rapid and standardized results. Widely deployed platforms, such as automated PCR or hybridization-based cartridges, have demonstrated robust performance for infectious disease diagnostics and AMR detection in both hospital and near-patient settings [60].

Cartridge-based platforms are particularly well suited for POC AMR diagnostics due to their ease of use, minimal operator training requirements, and compatibility with rapid clinical decision-making workflows. While these systems may be constrained by higher per-test costs and limited flexibility compared to open-format assays, their reliability, scalability, and clinical integration have supported widespread adoption in high-throughput and emergency diagnostic environments. Together, DNA microarrays and cartridge-based platforms complement isothermal and CRISPR-based approaches by offering high-level multiplexing and standardized implementation pathways for AMR surveillance and precision antimicrobial stewardship.

### 3.3. Immunological and Biosensor-Based Approaches

Immunological and biosensor-based approaches constitute an important class of diagnostic strategies that detect pathogens or AMR markers through specific molecular recognition events rather than nucleic acid amplification. These approaches typically rely on antigen–antibody interactions, receptor–ligand binding, or affinity-based sensing mechanisms to generate rapid and interpretable diagnostic signals. Their relatively simple workflows, fast turnaround times, and minimal instrumentation requirements have made them particularly attractive for POC diagnostics in both clinical and resource-limited settings [60].

Immunological assays, including enzyme-linked immunosorbent assays (ELISA), lateral flow immunoassays (LFIA), and agglutination tests, are widely used for the detection of pathogen-specific antigens, toxins, or host immune responses. Lateral flow assays, in particular, have achieved broad adoption due to their low cost, ease of use, and rapid visual readouts, often delivering results within minutes. In the context of AMR, immunological approaches have been applied to detect resistance-associated proteins, such as altered penicillin-binding proteins, carbapenemases, or specific bacterial toxins linked to resistant phenotypes [59].

Biosensor-based diagnostics extend these principles by integrating biological recognition elements with physical transducers that convert binding events into measurable electrical, optical, or mechanical signals. Common biosensor formats include electrochemical, optical (e.g., surface plasmon resonance), piezoelectric, and fluorescence-based sensors. These platforms enable highly sensitive and often quantitative detection of pathogens, resistance genes, or resistance-associated enzymes, with the potential for real-time monitoring and miniaturization [60].

Recent advances in nanomaterials, microfluidics, and surface chemistry have significantly enhanced the performance of biosensor-based diagnostics, improving sensitivity, specificity, and multiplexing capacity. Nanoparticle-enhanced sensors, for example, have been shown to lower detection limits and accelerate signal generation, while microfluidic integration enables automated sample handling and reduced reagent consumption. These innovations have expanded the applicability of biosensors for AMR detection at the POC, including rapid identification of resistant pathogens and monitoring of antimicrobial susceptibility [61].

Despite their advantages, immunological and biosensor-based approaches may face challenges related to limited sensitivity compared to nucleic acid amplification methods, potential cross-reactivity, and difficulty in distinguishing closely related resistance mechanisms. Nevertheless, their speed, operational simplicity, and compatibility with decentralized testing workflows position them as valuable complementary tools to molecular diagnostics. When combined with nucleic acid–based methods or deployed as frontline screening assays, immunological and biosensor-based platforms can significantly enhance rapid AMR detection and support timely clinical decision-making.

#### 3.3.1. Lateral Flow Assays

Lateral flow assays (LFAs) are among the most widely used immunological diagnostic tools for POC testing due to their simplicity, low cost, and rapid visual readout. LFAs operate on the principle of capillary-driven flow, in which liquid samples migrate along a porous membrane and interact with immobilized capture reagents, typically antibodies, resulting in the formation of visible test and control lines. Results are usually available within minutes, making LFAs highly suitable for decentralized and near-patient diagnostic applications [62].

In infectious disease diagnostics, LFAs have been extensively deployed for the detection of pathogen-specific antigens or host-derived biomarkers. In the context of AMR, LFAs have been developed to identify resistance-associated proteins, including β-lactamases, carbapenemases, and other enzymatic determinants that confer resistance to clinically important antibiotics. These assays enable rapid differentiation between susceptible and resistant strains, supporting early treatment decisions and infection control measures [63].

A key advantage of LFAs is their minimal operational complexity, requiring no specialized instrumentation or extensive sample preparation. Advances in label technologies, such as gold nanoparticles, latex beads, and fluorescent or magnetic reporters, have further improved assay sensitivity and expanded the range of detectable targets. Additionally, integration with smartphone-based readers and portable optical devices has enhanced result interpretation and data connectivity, increasing the clinical utility of LFAs in both high-resource and resource-limited settings [64].

Despite their strengths, LFAs generally exhibit lower analytical sensitivity compared to nucleic acid amplification methods and may face challenges related to cross-reactivity or limited multiplexing capacity. Nevertheless, their speed, affordability, and ease of deployment make LFAs valuable frontline screening tools for AMR detection at the POC. When used in combination with molecular confirmatory assays, LFAs can contribute significantly to rapid antimicrobial stewardship and effective patient management.

#### 3.3.2. Antibody- and Aptamer-Based Biosensors

The modern concept of biosensors dates back to the pioneering work of Clark and Lyons, who in 1962 described an enzyme-based electrode for measuring blood glucose [62]. Since then, biosensors have undergone substantial evolution, driven particularly by advances in nanotechnology, microfabrication, and material science. Contemporary biosensors are highly specialized analytical devices that couple a biological recognition element with a transducer capable of converting a biochemical interaction into a quantifiable signal [61,62]. Among these, antibody- and aptamer-based biosensors have attracted particular attention due to their high specificity, versatility, and suitability for POC applications.

**Figure 3:**
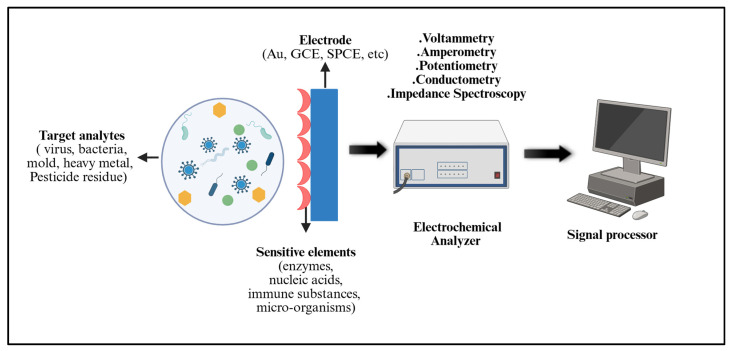
Fundamental components and signal transduction pathway of a typical electrochemical biosensor for target analyte detection. The schematic illustrates the core elements of an electrochemical biosensor, including target analytes (e.g., viruses, bacteria, fungi, heavy metals, and pesticide residues), biologically sensitive recognition elements (such as enzymes, nucleic acids, antibodies, immune substances, or whole microorganisms), and the transducer interface based on conductive electrodes (e.g., gold, glassy carbon, or screen-printed electrodes). Upon target recognition, biochemical interactions at the sensor surface generate an electrochemical signal that is processed by an electrochemical analyzer (e.g., impedance spectroscopy), converted into digital output, and subsequently displayed for data interpretation.

Biosensors are integral to diagnostics, environmental monitoring, food safety, and industrial process control. In healthcare, they enable the rapid, sensitive, and cost-effective detection of biomarkers and pathogens, supporting personalized medicine and antimicrobial stewardship [65]. A typical biosensor comprises five components (Figure 3): (i) the analyte of interest (for example, glucose, ammonia, nucleic acids, or whole cells); (ii) a bioreceptor, such as an enzyme, antibody, nucleic acid, cell, or aptamer, that specifically recognizes the analyte; (iii) a transducer that converts the biorecognition event into an electrical, optical, acoustic, or mechanical signal; (iv) associated electronics that process and amplify the signal; and (v) a display or read-out unit that presents the result in a user-friendly format [65].

Biosensors have demonstrated particular value in POC testing for AMR, as they can provide rapid pathogen identification and susceptibility information, thereby guiding appropriate antibiotic use and reducing unnecessary prescriptions [66]. They also support high-throughput screening of microbial responses to drugs, thereby informing the development of new antimicrobial strategies [67]. Major biosensor classes include electrochemical, acoustic wave, and optical biosensors, each offering distinct advantages and complementary capabilities.

##### Electrochemical Biosensors

Electrochemical biosensors detect changes in electrical properties, such as current, potential, and impedance, induced by biological recognition events occurring at the electrode surface. These devices are particularly attractive for AMR diagnostics because they can be miniaturized, integrated into portable platforms, and operated using relatively simple instrumentation [68].

Direct electrochemical detection often involves immobilizing whole microbial cells or specific recognition elements (antibodies, bacteriophages, or aptamers) onto an electrode. Binding of the target bacteria modulates electron transfer, which can be quantified using electrochemical techniques such as cyclic voltammetry, differential pulse voltammetry and impedance spectroscopy. Indirect strategies stimulate microbial cells to produce electroactive metabolites that serve as proxies for viability and drug susceptibility [68].

Nucleic acid-based electrochemical biosensors show potential promise for the detection of bacterial 16S rRNA, resistance genes and microRNAs. For example, Altobelli et al. (2017) reported an electrochemical platform that uses DNA capture probes to detect Enterobacteriaceae and quantify the effect of ciprofloxacin on bacterial growth within six hours, thereby bypassing the multiday culture protocols [67]. Also, Ranjbari et al. (2023) developed an electrochemical sensor for miRNA-122, a candidate biomarker in breast cancer, illustrating how similar architectures can be adapted for diverse clinical applications [69]. Although some examples originate from oncology and biomarker research, these architectures are directly translatable to AMR diagnostics.

Novel recognition elements enhance the selectivity and sensitivity of electrochemical biosensors. Jolly et al. (2016) designed an AptaMIP sensor for prostate-specific antigen (PSA) by embedding a thiolated DNA aptamer in a molecularly imprinted polymer layer on a gold electrode, achieving a three-fold sensitivity improvement over aptamer- or MIP-only formats using electrochemical impedance spectroscopy [70]. Li et al. (2018) proposed a microfluidic chip for detecting the insecticide carbofuran that combined a molecularly imprinted polymer with an aptamer-based read-out, attaining a low detection limit and demonstrating the potential for automated, high-throughput operation [71]. Zhang et al. (2020) described a dual-mode biosensor for cardiac troponin I that integrates molecular imprinting with antibody-functionalized Fe^3+^–polydopamine nanoparticles, enabling both electrochemical and colorimetric detection at clinically relevant concentrations [72].

Graphene field-effect transistors (G-FETs) represent an emerging class of electrochemical biosensors with excellent sensitivity, scalability, and biocompatibility [68]. Kumar et al. (2020) reported a G-FET platform functionalized with pyrene-conjugated peptides that can discriminate antibiotic-resistant from susceptible strains of Staphylococcus aureus and Acinetobacter baumannii. By applying an external electric field to enhance bacterial binding, the system delivered results within five minutes, underscoring the potential of G-FETs for rapid AMR diagnostics [73].

##### Acoustic Wave Biosensors

Acoustic wave biosensors exploit surface acoustic waves (SAWs) propagating along a piezoelectric substrate, such as SiO_2_ or LiNbO_3_, to detect changes in the mass or viscoelastic properties at the sensor surface. Interdigitated transducers (IDTs) generate and receive acoustic waves when alternating voltage is applied. When target molecules, such as bacteria or antigens, bind to biorecognition elements immobilized on the surface, they alter the wave velocity, frequency, or amplitude. These changes are converted into electrical signals that can be measured with high sensitivity [74].

An example of an antimicrobial application is the use of thin-film SAW devices on ZnO/Si platforms to modulate bacterial growth and inactivation. Ong et al. in 2024 showed that a low SAW power can promote the growth of *Escherichia coli* and *Staphylococcus aureus* through acoustic streaming and local heating, whereas a higher power results in bacterial inactivation through mechanical disruption and thermal effects. The incorporation of ZnO tetrapods enhanced these effects, indicating a synergistic route to physical and chemical antimicrobial strategies [75]. This dual functionality highlights the potential of acoustic wave platforms for both biosensing and antimicrobial intervention.

Acoustic-wave biosensors have also been developed for direct pathogen detection. In 2023, Gagliardi et al. reported a biosensor designed to target *Legionella pneumophila*, where antibodies immobilized on the sensor surface captured the bacterium, leading to measurable changes in acoustic properties. The device demonstrated high specificity against other organisms, such as *E. coli* and *Enterococcus faecium*, and delivered rapid results suitable for monitoring water systems and preventing outbreaks of Legionnaires’ disease [75]. Such rapid and label-free detection capabilities support the integration of acoustic wave biosensors into POC platforms for pathogen surveillance and AMR monitoring.

##### Optical Biosensors

Optical biosensors detect changes in optical properties associated with analyte binding, such as intensity, wavelength, phase, and polarization. They encompass a broad range of mechanisms including fluorescence, luminescence, surface plasmon resonance (SPR), localized surface plasmon resonance (LSPR), and colorimetric responses [76].

Fluorescent biosensors can provide high sensitivity and spatial resolution. Azad et al. (2021) developed luminescent biosensors based on bacterial luciferase to detect reactive oxygen species (ROS), providing a platform for high-throughput screening of drugs that modulate oxidative stress pathways [77]. Also, in 2023, Nikolaev et al. engineered a palette of 22 flavin-binding fluorescent proteins by mutagenesis of the light, oxygen and voltage (LOV) domain from *Chloroflexus aggregans*, enabling fine-tuned emission spectra between 486 and 512 nm. This toolkit allows multicolor imaging of bacterial and mammalian cells and illustrates how engineered fluorescent proteins can expand the capabilities of optical biosensing in microbiology and cell biology [78]. Such fluorescent platforms can be readily adapted for antimicrobial screening and resistance-associated phenotyping.

Colorimetric biosensors, particularly those based on plasmonic nanoparticles, are attractive for POC use because they provide visually interpretable results without the need for sophisticated equipment. Wenck et al. (2024) designed a gold nanoparticle-based colorimetric assay functionalized with Cetyltrimethylammonium Bromide (CTAB) and Monoethanolamine (MEA) to differentiate between oral bacteria, such as *Aggregatibacter actinomycetemcomitans*, *Actinomyces naeslundii*, *Porphyromonas gingivalis*, and *Streptococcus oralis*, via distinct color shifts. This approach demonstrated rapid and more specific bacterial detection with clear visual read-outs, underscoring the utility of LSPR-based biosensors in clinical and community settings [79].

Although optical biosensors offer high sensitivity and real-time monitoring, factors like the cost, stability, and integration into robust POC formats are still challenging. Ongoing advances in nanomaterials, photonics, and microfabrication are expected to further improve their performance, robustness, accessibility in healthcare, environmental monitoring and drug discovery fields. These advances support the translation of optical biosensors into POC platforms for rapid pathogen detection and AMR monitoring.

#### 3.3.3. Nanomaterial-Enhanced Sensing Platforms

Nanomaterial-enhanced sensing platforms have emerged as powerful tools for improving the performance of immunological and biosensor-based diagnostics by enhancing sensitivity, selectivity, and signal transduction efficiency. Nanomaterials offer unique physicochemical properties, including high surface-to-volume ratios, tunable optical and electrical characteristics, and versatile surface functionalization, which collectively enable more efficient biorecognition and signal amplification compared to conventional bulk materials [80].

A wide range of nanomaterials has been integrated into sensing platforms, including metallic nanoparticles (e.g., gold and silver), carbon-based nanostructures (graphene, carbon nanotubes, and carbon dots), metal–organic frameworks (MOFs), quantum dots, and magnetic nanoparticles. In biosensing applications, these materials can serve as signal enhancers, transducer modifiers, or carriers for antibodies, aptamers, enzymes, and nucleic acids. For example, gold nanoparticles are extensively used in colorimetric and plasmonic assays due to their strong localized surface plasmon resonance (LSPR), while graphene-based materials enhance charge transfer in electrochemical sensors, leading to lower detection limits and faster response times [81].

In the context of AMR diagnostics, nanomaterial-enhanced platforms enable rapid and sensitive detection of pathogens, resistance genes, and resistance-associated proteins at low concentrations. Nanoparticle-based amplification strategies have been applied to improve lateral flow assays, electrochemical biosensors, and optical sensors, facilitating the detection of resistant bacteria directly from complex clinical samples. Additionally, magnetic nanoparticles allow efficient target enrichment and separation, reducing background interference and improving assay robustness in POC settings [82,83].

Beyond signal enhancement, nanomaterials also support assay miniaturization, multiplexing, and integration with microfluidic systems, enabling compact and automated diagnostic devices. These features are particularly advantageous for decentralized testing environments, where rapid turnaround time, minimal sample processing, and operational simplicity are critical. However, challenges related to material reproducibility, long-term stability, scalability, and regulatory standardization must be addressed to ensure reliable clinical translation of nanomaterial-enhanced sensing platforms.

Overall, the integration of nanomaterials into biosensing technologies represents a key enabling strategy for next-generation POC diagnostics. By combining enhanced analytical performance with flexible platform design, nanomaterial-based sensing systems hold significant promise for improving rapid AMR detection, surveillance, and antimicrobial stewardship across diverse healthcare settings.

#### 3.3.4. Mobile-Based Platforms

Smartphones have emerged as versatile platforms for POC diagnostics because they combine high-resolution cameras, considerable computing power, intuitive user interfaces, and ubiquitous connectivity. When integrated with microfluidic chips, lateral-flow assays, or optical readers, smartphones can perform image acquisition, signal processing, and data transmission in a single device [82,83].

A smartphone-based monitoring approach can minimize hospital visits and reduce pressure on overcrowded healthcare facilities while maintaining continuity of care. They enable patients to perform self-testing at home or in the community, capture images of test results, and transmit data securely to healthcare providers for clinical interpretation. This connectivity facilitates rapid feedback, supports the remote monitoring of chronic infections, and allows the aggregation of anonymized data for public health surveillance. For patients undergoing long-term therapy or follow-up for infections with a risk of chronicity, smartphone-based monitoring approaches can minimize hospital visits and reduce pressure on overcrowded healthcare facilities while maintaining continuity of care [84]. Wood et al. (2019) evaluated a smartphone-connected microfluidic diagnostic platform in Rwanda for HIV and syphilis testing. In this study, healthcare workers collected 2 µL of whole blood from 96 patients and used a smartphone-microfluidic system to perform multiplex serological assays. The results were available within approximately 15 min, with reported sensitivities of 92–100% and specificities of 79–100%. Notably, 97% of participants expressed a preference for the smartphone-based approach compared with conventional testing, citing rapid turnaround and simplicity as major advantages [84]. These findings demonstrate the acceptability and feasibility of mobile-based diagnostics, particularly in low-resource and remote settings.

## 4. Commercially Available Point-of-Care Diagnostic Platforms for Antimicrobial Resistance

The translation of POC diagnostics for AMR from research laboratories to clinical and community settings has led to the development of several commercially available diagnostic platforms. These systems are designed to provide rapid, standardized, and user-friendly detection of pathogens and resistance determinants, enabling timely clinical decision-making and supporting antimicrobial stewardship initiatives. Unlike experimental assays, commercial POC platforms are typically validated for clinical use, integrated into routine workflows, and supported by regulatory approvals and quality control frameworks [84].

Most commercially available POC AMR platforms rely on nucleic acid–based technologies, particularly real-time PCR and isothermal amplification methods, integrated within closed and automated cartridge-based systems. These platforms combine sample preparation, amplification, detection, and result interpretation into a single disposable cartridge, minimizing hands-on time and reducing the risk of contamination. Results are often delivered within 30–90 min, making these systems suitable for emergency departments, intensive care units, and decentralized healthcare facilities [85].

Several commercial platforms have demonstrated strong performance in detecting key resistance genes, including those encoding carbapenemases, extended-spectrum β-lactamases (ESBLs), and methicillin resistance determinants. By enabling rapid identification of resistance profiles directly from clinical specimens, such platforms help shorten the time to appropriate therapy compared to conventional culture-based susceptibility testing, which can require several days. In addition to bacterial AMR, some platforms support multiplex detection of viral and fungal pathogens, further expanding their clinical utility [84].

Immunological and lateral flow–based commercial assays also play an important role in POC AMR diagnostics, particularly for the detection of resistance-associated enzymes and proteins. These assays offer extremely rapid turnaround times, often within minutes, and require minimal instrumentation. Although their analytical sensitivity may be lower than that of molecular platforms, they are valuable for frontline screening and triage, especially in low-resource or high-throughput settings.

Despite their advantages, commercially available POC AMR platforms face limitations related to cost, test menu flexibility, and coverage of emerging resistance mechanisms. Many systems are restricted to predefined panels, which may not capture novel or rare resistance genes. Additionally, the high per-test cost and reliance on proprietary consumables can limit widespread adoption in resource-limited regions. Nevertheless, continued technological innovation, expansion of target panels, and integration with digital health systems are expected to enhance the accessibility and impact of commercial POC AMR diagnostics in the coming years [86]. A translational overview of representative commercial and near-commercial POC platforms for AMR detection, including their regulatory status, evidence level, and potential stewardship role, is summarized in Table 2.

## 5. Clinical Applications and Impact

This section focuses on the real-world clinical deployment of POC diagnostics for AMR and their impact on patient management, antimicrobial stewardship, and healthcare systems. Rather than emphasizing analytical performance alone, the section examines how POC diagnostics influence clinical decision-making, prescribing behavior, and patient outcomes across different care settings. Particular attention is given to evidence from primary care, hospital, and resource-limited environments, as well as implementation factors that determine sustainable clinical impact.

### 5.1. Evaluation of Point-of-Care Diagnostics in Clinical Settings

POC diagnostics for pathogen identification and AMR are best evaluated using clinical utility outcomes rather than analytical novelty. In real-world practice, a test’s value depends on whether it delivers actionable information within a time window that changes management, improves patient outcomes, and supports antimicrobial stewardship. Key evaluation domains include: (i) diagnostic accuracy and agreement with reference standards (culture, AST, and validated molecular methods); (ii) turnaround time and time-to-result at the point of decision-making; (iii) impact on prescribing, including initiation, escalation/de-escalation, and duration of antibiotics; (iv) patient outcomes, such as complications, revisit rates, length of stay, and mortality in severe infections; and (v) implementation performance, including usability by intended operators, training requirements, quality control, connectivity, and integration with clinical workflows and stewardship programs. These domains align with how guidelines and target product profiles (TPPs) frame AMR diagnostics—emphasizing fitness-for-purpose, setting-specific needs, and measurable downstream effects rather than sensitivity alone [28].

Evidence for clinical benefit is strongest when POC testing is embedded within decision algorithms and stewardship interventions. In primary care, where a substantial proportion of antibiotics are prescribed empirically for respiratory syndromes, POC inflammatory marker testing has demonstrated stewardship benefits by reducing unnecessary antibiotic prescribing without compromising safety in appropriately implemented pathways. A meta-analysis of randomized and pragmatic studies found that C-reactive protein POC testing (CRP-POCT) significantly reduced immediate antibiotic prescribing compared with usual care (risk ratio ≈ 0.79), highlighting how a rapid test can influence prescribing behavior at the index consultation [87].

More recent real-world evaluations in general practice similarly report reductions in antibiotic prescriptions when CRP-POCT is implemented with clear guidance and clinician training [88,89]. In hospital settings, especially bloodstream infections and sepsis pathways, the clinical question is often not whether a target can be detected, but whether rapid identification and resistance information leads to earlier optimization of therapy. Studies consistently show that rapid diagnostics achieve the greatest impact when paired with active antimicrobial stewardship actions (e.g., real-time notification, pharmacist/ID review, protocolized de-escalation). A large network meta-analysis of studies in bloodstream infections reported that rapid diagnostic tests used in conjunction with stewardship programs were associated with improved outcomes, including reduced mortality compared with conventional approaches in many settings [90]. Consistent with this, hospital implementation studies of rapid blood-culture identification panels commonly report faster streamlining of therapy and improved stewardship process measures when stewardship teams intervene promptly on results [91].

Importantly, evaluation must be context-specific. In LMICs and other resource-limited settings, performance metrics should explicitly incorporate feasibility: robustness to environmental conditions, simplified sample handling, supply chain stability, affordability, and the ability to operate with limited laboratory infrastructure. In such settings, the clinical impact may be amplified because baseline access to timely culture and AST is often constrained; however, sustainability depends on training, quality assurance, and fit within care pathways [92].

The clinical evaluation of POC AMR diagnostics should move beyond “proof-of-detection” and emphasize implementation effectiveness, whether the test reliably changes prescribing decisions and outcomes at scale. This requires prospective clinical studies, standardized outcome definitions (time-to-appropriate therapy, de-escalation rates, antibiotic consumption metrics), and explicit linkage between test results and stewardship actions in the intended use setting [90]. The major clinical settings, evaluation outcomes, and implementation challenges relevant to POC diagnostics are summarized in Table 3.

### 5.2. Translational Challenges and Readiness of Novel Point-of-Care Diagnostics

Despite rapid innovation in POC diagnostics for pathogen identification and AMR detection, translation into routine clinical practice remains limited. This gap reflects not a lack of technological creativity, but the complex requirements for clinical readiness, including robust validation, interpretability of results, workflow integration, regulatory approval, and sustainable implementation. As a result, many novel POC platforms remain confined to laboratory demonstrations or small pilot studies, despite promising analytical performance.

A key determinant of translation is technology readiness level (TRL). Many emerging POC diagnostics for AMR operate at early to intermediate TRLs, where feasibility and analytical validity have been demonstrated, but prospective clinical validation is insufficient. Moving toward routine use requires large-scale studies comparing POC results against reference culture-based AST, assessing concordance, failure modes, and patient safety across heterogeneous clinical populations. Without such evidence, even analytically robust platforms face barriers to regulatory approval and clinical acceptance [23,24].

Another major translational challenge relates to the clinical interpretability of resistance signals. Genotypic POC assays can rapidly identify resistance genes or mutations; however, the presence of a resistance determinant does not always equate to phenotypic resistance, particularly when gene expression is regulated, inducible, or context-dependent. Conversely, rapid phenotypic assays may fail to detect low-level or emerging resistance mechanisms. These discordances complicate clinical decision-making and reinforce the need to position many POC tests as decision-support tools rather than standalone determinants of therapy [25].

Operational and workflow integration barriers further limit readiness. Many novel POC diagnostics rely on specialized consumables, controlled storage conditions, or external instrumentation that hinder deployment in decentralized environments. In addition, lack of integration with electronic health records, laboratory information systems, and antimicrobial stewardship workflows can substantially reduce clinical impact. Evidence consistently shows that rapid diagnostics improve outcomes only when results are acted upon promptly, often through structured stewardship interventions rather than passive reporting alone [90].

Regulatory and economic considerations also shape translational readiness. Because AMR diagnostics directly inform therapeutic decisions, regulatory agencies often require evidence extending beyond analytical accuracy to include clinical performance and risk mitigation. At the same time, reimbursement models in many health systems do not adequately reflect the downstream value of diagnostics in reducing inappropriate antibiotic use, shortening hospital stays, or limiting resistance emergence. This misalignment discourages investment and slows adoption of otherwise promising POC technologies [27].

**Figure 4:**
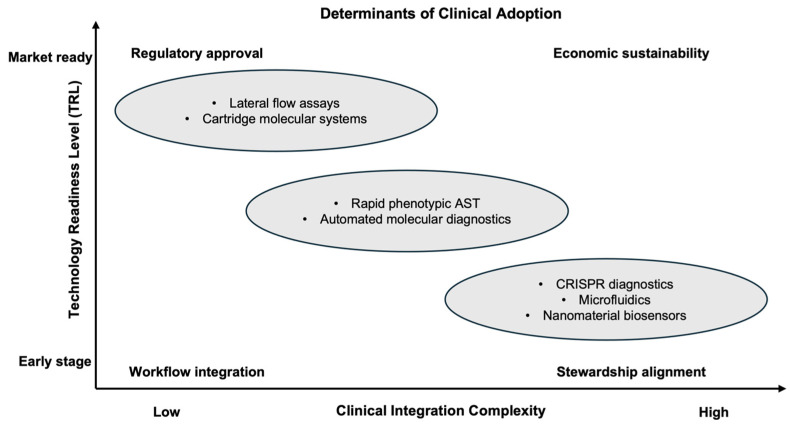
Translational landscape of POC diagnostic technologies according to technology readiness and clinical integration complexity. A two-dimensional conceptual matrix mapping current and emerging POC diagnostic platforms based on relative technology readiness level (TRL) and degree of clinical integration complexity. Established technologies, such as lateral flow assays and cartridge-based molecular systems, demonstrate high readiness and operational simplicity. Rapid phenotypic AST and automated molecular diagnostics occupy intermediate translational stages. Emerging platforms which include CRISPR-based diagnostics, advanced microfluidic systems, and nanomaterial-enhanced biosensors, remain at earlier developmental phases and are associated with higher integration complexity. Key determinants influencing clinical adoption: including regulatory approval pathways, economic sustainability, workflow integration, and alignment with antimicrobial stewardship frameworks, which are highlighted as critical translational barriers.

Taken together, translational readiness of novel POC diagnostics depends less on incremental gains in analytical sensitivity and more on clinical validation, interpretability, workflow compatibility, and system-level incentives. Future development efforts are increasingly prioritizing hybrid diagnostic strategies, stewardship-linked deployment models, and evaluation frameworks centered on clinically meaningful outcomes such as time-to-appropriate therapy and antibiotic optimization. Aligning innovation with these translational requirements will be essential for novel POC diagnostics to achieve sustained clinical and public health impact in AMR management [28]. The translational positioning of current and emerging POC technologies according to technology readiness and clinical integration complexity is presented in Figure 4.

### 5.3. Impact on Patient Outcomes and Antimicrobial Resistance Management

Bacterial infections continue to impose substantial morbidity, mortality, and economic burden worldwide, and AMR further limits effective treatment options. Traditional culture-based diagnostics and AST commonly require one to several days to deliver actionable results, which often leads clinicians to initiate broad-spectrum empiric therapy while awaiting confirmation. In this context, POC and near-patient rapid diagnostics can improve care only if they shorten the time to clinically meaningful actions, such as targeted therapy initiation, timely de-escalation, or avoidance of unnecessary antibiotics [90].

The strongest evidence of outcomes in acute care comes from bloodstream infection pathways where rapid diagnostic testing is combined with antimicrobial stewardship interventions. A large systematic review and network meta-analysis (88 studies; >25,000 patient encounters) found that rapid diagnostic tests integrated with antimicrobial stewardship programs were associated with improved clinical outcomes, including a survival benefit compared with conventional approaches in many settings. This work reinforces a key implementation principle: the benefit of rapid diagnostics is maximized when results are translated rapidly into treatment decisions through structured stewardship workflows rather than passive reporting alone [90]. Supporting real-world studies of blood culture identification panels similarly show improvements in stewardship-relevant process outcomes such as reduced time to optimal therapy and earlier de-escalation of broad-spectrum agents after implementation [92,93].

In community and ambulatory settings, the most consistent and measurable clinical impact relates to reducing inappropriate antibiotic prescribing, particularly for respiratory tract infections where diagnostic uncertainty is high. A systematic review of CRP POC testing in primary care reported a significant reduction in immediate antibiotic prescribing for respiratory tract infections, without evidence of worse clinical recovery outcomes, although some studies reported modest increases in re-consultation rates [91]. Evidence from pragmatic evaluations in other care contexts also supports the conclusion that CRP testing, when paired with clear guidance, can safely reduce antibiotic prescribing [94,95]. Collectively, these findings indicate that rapid POC testing can contribute to AMR mitigation by lowering unnecessary antibiotic exposure and therefore reducing selection pressure at the population level—provided that tests are embedded within prescribing algorithms and clinician training.

Beyond individual patient management, rapid and POC diagnostics can strengthen AMR control through improved infection prevention and surveillance. Faster identification of pathogens and resistance patterns can support earlier isolation precautions, outbreak recognition, and more responsive stewardship interventions. However, realizing these system-level benefits depends on connectivity (reporting and data capture), standardized interpretation, and sustainable implementation models that align incentives across laboratories, clinicians, and health systems [94,96].

The evidence base indicates that rapid diagnostics can improve patient care and AMR management through two dominant pathways: (i) earlier optimization of antimicrobial therapy in severe infections when paired with stewardship action, and (ii) reductions in unnecessary antibiotic prescribing in outpatient care when paired with clinical decision support. The magnitude of benefit varies by setting and implementation quality, emphasizing that impact should be evaluated using outcomes such as time to appropriate therapy, de-escalation rates, antibiotic consumption metrics (e.g., days of therapy [DOT]/defined daily doses [DDD]), length of stay, and mortality where applicable, rather than analytical performance alone [87,93,94]. The major implementation strategies recommended to mitigate antimicrobial resistance and support stewardship integration are summarized in Table 4.

## 6. Challenges and Limitations of Point-of-Care Diagnostics for Antimicrobial Resistance

POC diagnostics for AMR hold considerable promise, but face several interrelated challenges that must be addressed to realize their full potential. These challenges can be broadly grouped into analytical performance, economic and health-system constraints, and regulatory and implementation barriers.

From an analytical standpoint, the sensitivity, specificity, and overall accuracy are critical determinants of clinical utility. POC tests must be capable of detecting low concentrations of bacteria or resistance genes in complex samples, such as blood, urine, or respiratory secretions. Insufficient sensitivity increases the risk of false-negative results, delayed or inappropriate therapy, and poor outcomes [25,97]. Sensitivity is influenced not only by assay chemistry and device design, but also by disease prevalence, pathogen load at the time of sampling, and pre-analytical factors such as sample handling [96].

High specificity is equally important for minimizing false positives and avoiding unnecessary antibiotic exposure. Cross-reactivity with non-target organisms or host components can undermine specificity, particularly in syndromic panels or multiplexed assays [101,104]. Achieving and maintaining high specificity requires careful selection and validation of biorecognition elements, robust assay optimization, and rigorous clinical performance studies. Accuracy, which reflects the combined effect of sensitivity and specificity, must be demonstrated across diverse populations and clinical settings to ensure reliable performance [24,105].

Economic considerations pose a substantial barrier to the widespread implementation of POC. The costs associated with POC diagnostics include not only the price of test cartridges or consumables, but also staff time, training, quality assurance, instrument maintenance, and data management [104,105]. In many health systems, the reimbursement frameworks are not aligned with the true costs or benefits of POC testing. Prices are often determined by market forces rather than regulated by authorities, and fixed tariffs may not accommodate innovative, but initially more expensive, tests [106]. Insufficient or poorly targeted funding discourages healthcare providers from adopting POC tests, especially when there is no dedicated remuneration for performing the test.

Furthermore, conventional cost-effectiveness analyses frequently fail to capture the broader societal costs of AMR, including prolonged hospital stays, long-term disability, and productivity losses [107]. While several studies have shown that POC tests can reduce antibiotic prescription and hospitalization, leading to potential long-term savings, these benefits may be diffused and accrue outside the budget lines responsible for purchasing diagnostics [12]. This misalignment can hinder investment in POC technologies. For the industry, uncertainty about return on investment (ROI) and complex regulatory requirements can discourage the development of new POC diagnostics [96,97,100,101,102,104,105,106,107,108,109,110].

**Figure 5:**
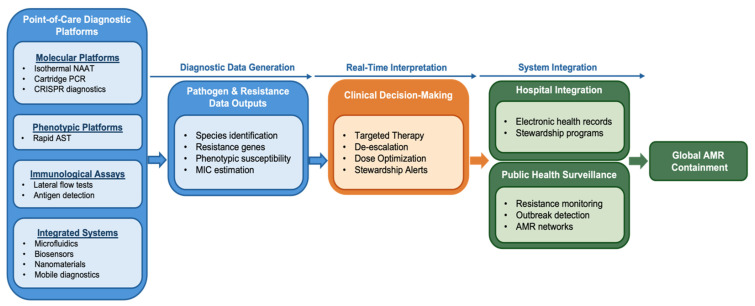
Integrated ecosystem model of point-of-care (POC) diagnostics for antimicrobial resistance (AMR). Schematic representation of the translational continuum linking diverse POC diagnostic technologies to clinical and public health impact. POC platforms; including rapid phenotypic antimicrobial susceptibility testing (AST), isothermal nucleic acid amplification tests (NAATs), CRISPR-based detection systems, immunological assays, biosensors, microfluidic devices, nanomaterial-enhanced platforms, and mobile-integrated technologies which generate actionable pathogen and resistance data (e.g., species identification, resistance determinants, and phenotypic susceptibility profiles). These outputs inform real-time clinical decision-making, enabling targeted antimicrobial selection, dose optimization, de-escalation strategies, and stewardship interventions. At the systems level, the integrated system with electronic health records (EHRs), hospital antimicrobial stewardship programs, and regional or global surveillance networks facilitates resistance trend monitoring and outbreak detection, ultimately strengthening coordinated AMR containment efforts.

Regulatory pathways for POC diagnostics are complex and increasingly demanding. In the United States, POC tests are regulated by the Food and Drug Administration (FDA) under the Federal Food, Drug, and Cosmetic Act, with additional mechanisms, such as emergency use authorizations (EUAs), during public health emergencies [111]. In Europe, the In Vitro Diagnostic Regulation (IVDR) and Medical Device Regulation (MDR) implement stricter requirements for performance evaluation, post-market surveillance, and risk classification [103]. Globally, the World Health Organization (WHO) provides guidance through frameworks such as the ASSURED criteria, affordable, sensitive, specific, user-friendly, rapid and robust, Equipment-free, and Deliverable to aid the design and deployment of diagnostics, particularly in low-resource settings [112]. International bodies, including the Global Harmonization Task Force and the International Medical Device Regulators Forum, aim to harmonize regulatory expectations, but navigating these evolving frameworks remains complex for innovators [113,114]. The integrated ecosystem model linking POC diagnostics to clinical decision-making, stewardship programs, and AMR surveillance networks is illustrated in Figure 5.

## 7. Future Directions and Emerging Trends

This section explores future directions and emerging trends that will shape the development, deployment, and impact of POC diagnostics for AMR. Building on the clinical evidence, translational challenges, and implementation barriers discussed in previous sections, it focuses on strategies required to move POC diagnostics from promising technologies to sustainable components of healthcare systems. Particular emphasis is placed on system-level integration, workforce capacity building, and policy frameworks needed to support scalable, equitable, and clinically meaningful use of POC diagnostics across diverse care settings. The integrated ecosystem model linking POC diagnostics to clinical decision-making, stewardship programs, and AMR surveillance networks is illustrated in Figure 4.

### 7.1. Strategies for Integration into Healthcare Infrastructure

The effective integration of POC diagnostics for AMR into healthcare systems requires coordinated strategies that address both clinical and organizational dimensions. Antimicrobial stewardship programs should explicitly incorporate rapid diagnostics into guidelines for empirical and targeted therapy, ensuring that test results are used to refine antibiotic selection, dosage, and duration [115]. Infection prevention and control measures, such as screening protocols for multidrug-resistant organisms, isolation policies, and environmental decontamination, can be strengthened by timely diagnostic information that can identify colonized or infected patients earlier [116].

Healthcare system planners should consider POC diagnostics when designing patient pathways, laboratory networks, and information systems. Integration with electronic health records and surveillance platforms enables real-time reporting of results and facilitates the aggregation of data for local and national AMR surveillance [117]. Investment in robust supply chains, maintenance support, and quality assurance systems is essential to ensure the sustained, reliable operation of POC platforms. Research and development incentives should encourage innovation in assays tailored to the epidemiological and infrastructural realities of different regions [118,119].

### 7.2. Training and Capacity-Building Needs

Training and capacity building are critical for the successful deployment of POC diagnostics. Healthcare professionals must understand both the technical aspects of test operations and clinical interpretation of results in the context of patient management. Educational programs should emphasize the mechanisms of AMR, principles of antimicrobial stewardship, and appropriate use of POC tests within diagnostic and treatment algorithms [120,121].

Laboratory staff and clinicians in resource-limited settings often face additional constraints including high workloads and limited access to continuing professional development. Capacity-building initiatives should prioritize these settings, offering modular, context-appropriate training that covers specimen collection, biosafety, quality control, and troubleshooting [122]. Interdisciplinary training models that combine clinicians, microbiologists, pharmacists, and public health practitioners can foster more effective, system-wide responses to AMR. Remote learning tools and digital platforms may further extend the reach of training.

### 7.3. Policies to Promote Point-of-Care Diagnostics for Antimicrobial Resistance Management

Policy frameworks have a crucial role in the development of an enabling environment for POC diagnostics. National action plans on AMR need to explicitly recognize the contribution of rapid diagnostics to stewardship and infection control, ensuring proper resource allocation is made to support this [123,124]. Reimbursement policies should reflect the value that accurate, rapid diagnostics bring to improved patient outcomes and reduction in inappropriate antibiotic use in order to incentivize uptake. Procurement policies should give preference to those assays that meet established performance and quality criteria and are suitable for intended use environments.

In high-income countries, this may involve policy efforts aimed at clearer regulatory pathways, targeted funding mechanisms, and integration into digital health infrastructures that support innovative diagnostic technologies [27]. In LMICs and sub-Saharan Africa, POC or near-POC rapid diagnostic tests that include antibiotic susceptibility information could substantially strengthen healthcare delivery where long travel distances and limited access to laboratory services impede follow-up and timely treatment [92,125]. Sustained adoption of these technologies will depend on maintaining their affordability, ensuring a reliable supply chain, and providing implementation support that is appropriate to the specific operational context.

### 7.4. Role of Point-of-Care Diagnostics in Global Antimicrobial Resistance Surveillance

POC diagnostics have the potential to play a transformative role in global AMR surveillance by enabling timely, decentralized, and data-rich detection of resistant pathogens across diverse healthcare settings. Traditional AMR surveillance systems rely heavily on centralized laboratory infrastructure, culture-based AST, and delayed reporting, which can limit representativeness and responsiveness—particularly in LMICs. In contrast, POC diagnostics can generate resistance-relevant data closer to the patient, supporting earlier detection of emerging resistance patterns and reducing geographic and temporal blind spots in surveillance networks.

When integrated with digital reporting and connectivity platforms, POC diagnostics can contribute real-time or near–real-time data to local, national, and global AMR surveillance systems. Automated data capture, standardized result interpretation, and secure transmission to public health databases enable aggregation of resistance trends across facilities and regions. Such capabilities align with the objectives of global surveillance initiatives, including the World Health Organization’s Global Antimicrobial Resistance and Use Surveillance System (GLASS), which emphasizes standardized data collection, representativeness, and timely reporting to inform policy and public health action [126].

POC diagnostics are particularly valuable in under-resourced and remote settings where access to centralized microbiology laboratories is limited. In these contexts, even basic resistance or pathogen identification data can substantially enhance situational awareness, guide empiric treatment guidelines, and support outbreak detection. Mobile- and cartridge-based POC platforms, when coupled with minimal training and robust quality assurance frameworks, offer a pragmatic pathway to expand surveillance coverage beyond tertiary care centers and into primary care, community clinics, and field settings [98].

Despite their promise, the contribution of POC diagnostics to AMR surveillance depends on several critical factors. These include harmonization of test outputs with surveillance definitions, data quality assurance, interoperability with existing health information systems, and clear governance frameworks for data ownership and use. Without standardized reporting structures and oversight, fragmented POC data may be underutilized or excluded from formal surveillance efforts. Therefore, surveillance-oriented deployment of POC diagnostics should be designed in coordination with public health authorities, ensuring alignment with national AMR action plans and global reporting standards [127].

In summary, POC diagnostics can complement traditional laboratory-based surveillance by improving the timeliness, granularity, and geographic reach of AMR data. As digital health infrastructures mature and connectivity improves, strategically deployed POC diagnostics have the potential to strengthen global AMR surveillance, support early warning systems, and inform more responsive and equitable public health interventions. Despite significant technological advances, the clinical implementation of POC diagnostic platforms requires careful evaluation of reproducibility, variability across clinical settings, and potential sources of bias in diagnostic performance. Differences in patient populations, sample handling, and testing conditions may influence outcomes, highlighting the need for standardized validation frameworks and cautious interpretation of diagnostic results in real-world settings [99]. Furthermore, integrating diagnostic results with clinical context and antimicrobial stewardship frameworks is essential to ensure appropriate interpretation and effective translation into clinical decision-making.

## 8. Conclusions

A coordinated global response against AMR urgently requires rapid, accurate, and decentralized diagnostics. Advances in isothermal amplification technologies, microfluidics, biosensing, and mobile platform development have opened new opportunities for POC pathogen and resistance marker detection bringing advanced diagnostic capabilities closer to the patient and the clinician. Miniaturized device-integrated optical, electrochemical, acoustic, and plasmonic biosensors enable highly sensitive detection with ever-shortening turnaround times. Smartphone-enabled systems allow for remote monitoring and real-time data sharing across both healthcare and public health networks.

Most of the promising technologies are still at the prototype stage and need further optimization, extensive clinical validation and sound economic evaluation. Despite this progress, analytical performance must be balanced against simplicity and robustness, particularly in resource-constrained settings. Economic, regulatory, and implementation obstacles persist; coherent policy, proper reimbursement models and investment in training and infrastructure remain underdeveloped.

Successful implementation of POC diagnostics for AMR will necessitate collaboration between clinicians, microbiologists, engineers, health economists, policymakers, and industry. If incorporated into clinical pathways and public health strategies, these technologies have the potential to accelerate appropriate use of antibiotics and improve patient outcomes and make an important contribution to containing antimicrobial resistance.

## Figures and Tables

**Table 1:** Conceptual comparison of phenotypic and genotypic point-of-care (POC) diagnostics for antimicrobial resistance (AMR).

Feature	Phenotypic POC Diagnostics	Genotypic POC Diagnostics
Measurement principle	Functional response to antibiotics	Detection of resistance genes or mutations
Detection principle	Measures bacterial growth, metabolism, morphology, or viability in the presence of antibiotics	Identifies specific nucleic acid sequences associated with resistance using amplification, hybridization, or sequencing
Requirement for viable cells	Typically, yes	No
Turnaround time	Hours (growth- or metabolism-dependent)	Minutes to hours (amplification-dependent)
Sensitivity/Specificity	Generally high for expressed resistance, but may vary depending on organism growth conditions and assay design	Typically high for known resistance markers, but limited by the genetic targets including the assay
Ability to detect unknown resistance	Yes (if phenotypically expressed)	No (limited to known determinants)
Clinical readiness level	Several methods already integrated into clinical microbiology workflows, but true rapid POC implementation remains limited	More advanced for POC use, with several commercially available assays already implemented in clinical settings
Implementation	Need for viable organisms, culture dependence, slower turnaround for slow-growing pathogens, and difficulty miniaturizing assays for bedside use	Limited detection of emerging or unknown resistance mechanisms, higher equipment cost, need for molecular expertise, and possible genotype-phenotype discordance
Typical limitations	Growth dependence; slower for slow-growing organisms	Incomplete resistance coverage; genotype–phenotype discordance

**Table 2:** Translational Landscape of Representative Commercial and Near-Commercial POC Platforms for AMR Detection. Platforms are categorized according to technology class and translational maturity. Key references correspond to the numbered reference list of the manuscript.

Platform/Developer	Technology Category	Diagnostic Target	Evidence Level	Regulatory Status	Time to Result	Potential Role in AMR	Key References
QuickMIC (Gradientech)	Rapid phenotypic AST (microfluidic MIC gradient)	Bloodstream infections (Gram-negative bacteria)	Peer-reviewed validation studies	CE-marked (EU)	2–4 h	Early targeted therapy and antimicrobial optimization	[11,74,75]
Captiver (Astrego Diagnostics/Sysmex)	Single-cell microfluidic growth-based AST	UTI and bloodstream infections	Laboratory and translational validation	Under clinical deployment	<1 h (ID), 2–6 h (AST)	Rapid susceptibility-guided antibiotic selection	[3,7]
Coris BioConcept (Carbapenemase LFAs)	Immunochromatographic lateral flow assay	Carbapenemase-producing Enterobacterales	Clinical validation studies	CE-marked	15–30 min	Rapid resistance enzyme detection for infection control	[57]
SmartWound (University of Bath Consortium)	Fluorescent toxin detection (wound dressing-based)	Wound pathogens (*S. aureus*, *P. aeruginosa*, *Candida* spp.)	Patent + prototype validation	Translational prototype	~15 min	Rapid wound infection triage and monitoring	[73]
Smartphone-integrated Microfluidic Platforms	Mobile-integrated molecular or immunoassay systems	Multiplex infectious disease targets	Peer-reviewed field validation	Research/selective deployment	15–30 min	Decentralized diagnostics and surveillance support	[24,25]
Inspector-01 (Spectral Platforms)	Raman spectroscopy-based metabolic profiling	Bloodstream infection stress signatures	Prototype/limited validation	Pre-commercial	~30 min	Early pathogen stress response detection	[65]

**Table 3:** Clinical evaluation framework for point-of-care (POC) diagnostics in pathogen identification and antimicrobial resistance (AMR) detection.

Clinical Setting	Primary Clinical Question	Key Evaluation Outcomes	Typical Benefits Observed	Common Limitations
**Primary care**	Is an antibiotic indicated at this visit?	Reduction in immediate antibiotic prescribing; safety outcomes (reconsultation, complications); clinician adherence to guidance	Decreased unnecessary antibiotic use; improved clinician–patient communication	Limited pathogen specificity; reliance on decision algorithms and training
**Emergency department**	Should empiric therapy be initiated or withheld?	Time to treatment decision; concordance with reference diagnostics; patient flow metrics	Faster triage and targeted initiation of therapy; reduced diagnostic uncertainty	Variable impact without integration into care pathways
**Hospital wards**	Can therapy be optimized or de-escalated earlier?	Time to appropriate therapy; de-escalation rates; antibiotic consumption metrics (DDD/DOT)	Earlier streamlining of therapy; improved stewardship outcomes	Benefit depends on timely stewardship intervention
**Intensive care units**	Is broad-spectrum coverage still required?	Time to de-escalation; length of stay; mortality in severe infections	Improved antimicrobial optimization in high-risk patients	High cost; complexity of implementation
**Low- and middle-income settings**	Can actionable diagnostics be delivered without centralized labs?	Feasibility; robustness; time to actionable result; linkage to treatment	Improved access to diagnostics; reduced diagnostic delays	Supply chain stability; training and quality assurance
**Public health and surveillance**	Can resistance trends be detected earlier?	Data completeness; reporting timeliness; integration with surveillance systems	Enhanced outbreak detection and resistance monitoring	Data standardization and connectivity challenges

**Table 4:** Summary of recommended implementation strategies to mitigate antimicrobial resistance (AMR).

Implementation Strategy	Description	Reference
**Antibiotic Stewardship Programs**	Initiatives designed to promote the appropriate use of antibiotics, including guidelines for prescribing.	[96,97]
**Enhanced Surveillance Systems**	Development of national and international surveillance systems to monitor AMR patterns and antibiotic use.	[91,98,99]
**Point-of-Care (POC) Diagnostics**	Implementation of rapid diagnostic tests at the point of care to guide appropriate antibiotic therapy especially in LMIC.	[92,100,101,102]
**Infection Prevention and Control (IPC) Measures**	Establishing protocols to prevent infections in healthcare settings, reducing the need for antibiotics.	[95]
**Education and Training**	Ongoing education for healthcare professionals and the public on the risks of AMR and the importance of stewardship.	[96,103]
**Regulatory Policies on Antibiotic Use**	Development of regulations to restrict the use of antibiotics in agriculture and promote responsible prescribing.	[89,90]
**Research and Development of New Antibiotics**	Funding and support for the discovery and development of new antimicrobial agents and alternatives.	[87,88]

## Data Availability

Data sharing is not applicable to this article as no new data were created or analyzed.
